# A rare occurrence of bilateral C-shaped roots in mandibular first and second premolars diagnosed with the aid of spiral computed tomography

**DOI:** 10.4317/jced.51459

**Published:** 2014-10-01

**Authors:** Raju Chauhan, Shweta Singh, Anil Chandra

**Affiliations:** 1 Reader, BDS, MDS. Department of Conservative Dentistry and Endodontics, Saraswati Dental College and Hospital, Lucknow, Uttar Pradesh, India; 2 Senior Lecturer, BDS, MDS. Department of Oral and Maxillofacial Pathology, Saraswati Dental College and Hospital, Lucknow, Uttar Pradesh, India; 3 Professor, BDS, MDS. Department of Conservative Dentistry and Endodontics, Faculty of Dental Sciences, King Georges Medical University, Lucknow, Uttar Pradesh, India

## Abstract

The C-shaped canal system is an anatomical variation mostly seen in mandibular second molars, although it can also occur in maxillary and other mandibular molars. Such variation in the root canal anatomy is uncommon in mandibular first premolars and very rare in mandibular second premolars. The C-shaped canal is so named for the cross-sectional morphology of the root and root canal. The main anatomic feature of C-shaped canals is the presence of a fin or web connecting the individual root canals. Failure of the Hertwig’s epithelial root sheath to fuse on the lingual or buccal root surface is the main cause of C-shaped roots, which always contain a C-shaped canal. This case report describes an unusual occurrence of C-shaped roots in mandibular first and second premolars bilaterally, diagnosed with the aid of spiral computed tomography.

** Key words:**Canal configuration, C-shape, mandibular first premolar, mandibular second premolar, spiral computed tomography scans.

## Introduction

The C-shaped canal system is characterized by the presence of a C-shaped groove that communicates with one or more canals in cross section. There are many variations of the root canal system in these types of teeth along the length of the root (1). The most important anatomical feature of C-shaped canals is the presence of a fin or web connecting the individual root canals. Typically, this type of root canal system is found in teeth with fusion of roots either buccally or lingually. These complex root canal configurations can be difficult to recognize, prepare and obturate (2). The C-shaped canals occur more frequently in mandibular second molars (3) although it is also found in mandibular first molars, maxillary molars, mandibular premolars and maxillary lateral incisors.

The incidence of C-shaped canals in mandibular first premolars is 10-18% (4), but no case report of this canal configuration has been identified in mandibular second premolars (4,5). Cooke and Cox (6) in 1979 indicated that it would be difficult to recognize the existence of C-shaped canals on the basis of radiograph alone. The best method for accurate determination of the root canal morphology of a tooth is serial sectioning of the tooth, which is not feasible in clinical situations. Therefore, other diagnostic methods like spiral computed tomography [CT] are useful in clinically to determine the root canal morphology.

This case report describes the rare occurrence of C-shaped root canal anatomy bilaterally in mandibular first and second premolars diagnosed with the aid of spiral CT.

## Case Report

A 24 yrold male patient with a noncontributory medical history reported to our hospital with the chief complaint of pain in lower right back teeth for the past 3-4 months and food lodgment/cold sensitivity in lower left back teeth. Clinical examination revealed deep carious lesions in tooth #46 and #36. Tooth #46 was tender on percussion whereas tooth #36 was asymptomatic. Sensibility testing [cold test and EPT] on both the teeth revealed the non-vitality in tooth #46 while tooth #36 was vital.

**Figure 1:**
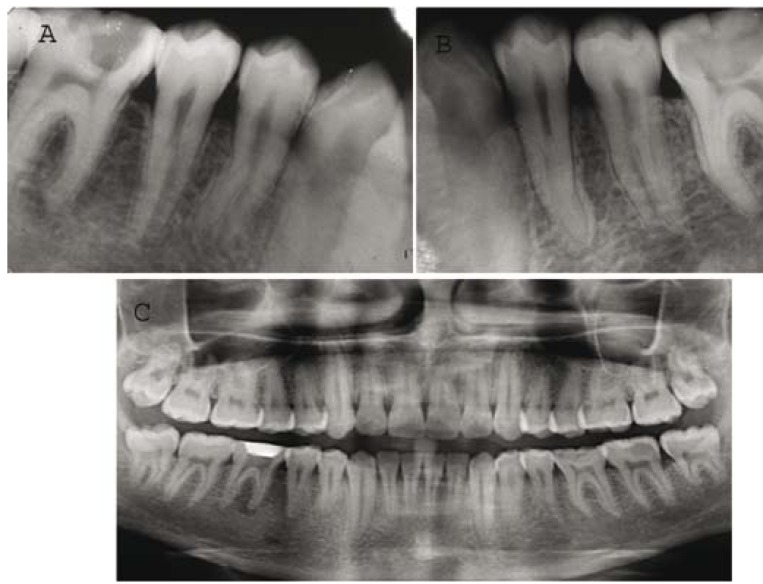
A) Pre operative radiograph revealing unusual root canal anatomy of tooth #44 and 45; B) Pre operative radiograph revealing unusual root canal anatomy of tooth #35 and 34; C) Panoramic radiograph illustrating some form of abnormal root and root canal system morphology associated with each of the mandibular premolars.

Periapical radiographs revealed deep carious lesion and periradicular radiolucency in relation to tooth #46 (Fig. 1). Occlusal caries was also present in tooth #36 but periradicular changes were not evident (Fig. 1). Single visit root canal treatment was done in tooth #46 with crown down technique and tooth #36 was restored with composite resin [3M ESPE Dental Products, St Paul, MN].

While examining the periapical radiographs of both mandibular first molars [tooth #46 and #36], unusual and complex root canal anatomy were observed in all mandibular premolars (Fig. 1). At least two roots were visible radiographically. Panoramic radiograph was taken to identify similar variations in the root canal anatomy/morphology related to other teeth in the oral cavity (Fig. 1).

Radiographs revealed the presence of at least two roots in mandibular premolars bilaterally (Fig. 1) but did not provide any clue regarding the three-dimensional structure and internal morphology of the root canals. Conventional radiography has an inherent disadvantage of providing a two-dimensional image of a three-dimensional object resulting in superimposition of images.

**Figure 2:**
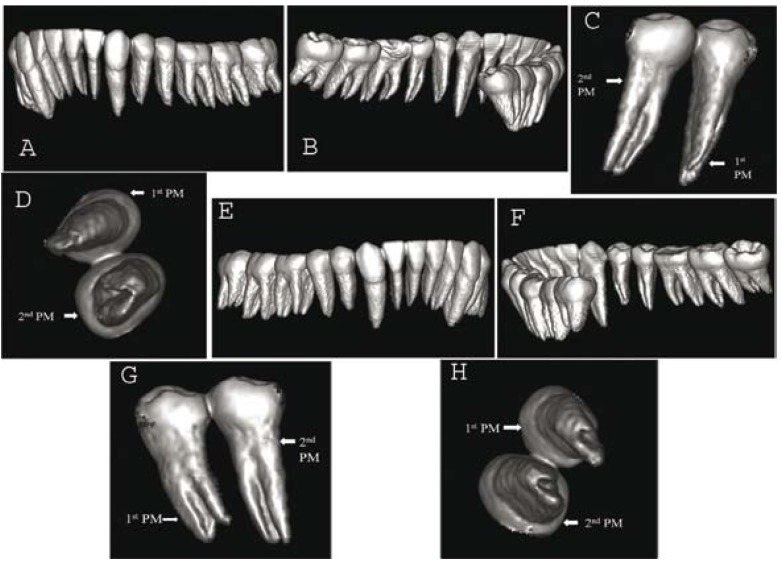
Spiral CT images; A) Three-dimensional reconstruction of the mandible (left posterior buccal view); B) Three-dimensional reconstruction of the mandible (left posterior lingual view); C) Three-dimensional reconstruction of tooth #35 and #3421 (lingual view); D) Three-dimensional reconstruction of tooth #35 and #34 (inferior view); E) Three-dimensional reconstruction of the mandible (right posterior buccal view); F) Three-dimensional reconstruction of the mandible (right posterior lingual view); G) Three-dimensional reconstruction of tooth #44 and #45 (lingual view); H) Three-dimensional reconstruction of tooth #44 and #45 (inferior view).

**Figure 3:**
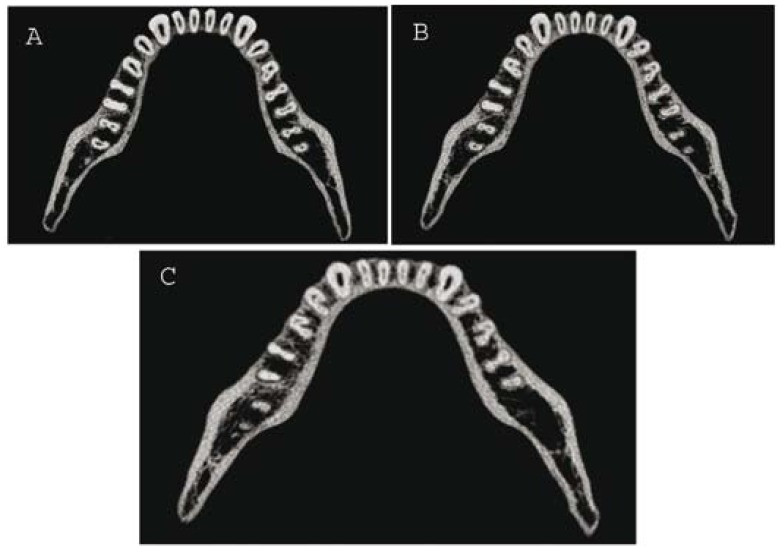
Axial CT slices; A) Coronal third section of the roots of mandibular premolars; B) Middle third section of the roots of mandibular premolars; C) Apical third section of the roots of mandibular premolars.

To ascertain the three-dimensional morphology of all mandibular premolars, dental imaging with the help of spiral CT was therefore planned. After obtaining an informed consent from the patient spiral CT of the mandible was performed by using the dental software Dentascan [GE Healthcare, Milwaukee, WI]. The parameters used were: tube voltage, 120 KVp; tube current, 100 mAs; displayed field of view [DFOV], 17 cm; slice thickness, 0.6 mm. The morphology of all mandibular premolars was achieved with three-dimensional reconstructed images (Fig. 2). The mandibular premolars were also focused to get the axial cross-sections of 0.6 mm thickness (Fig. 3).

The reconstructed images of SCT revealed an unusual and complex root canal anatomy in all mandibular premolars. C-shaped root canal anatomy was present in mandibular premolars bilaterally (Fig. 2). A deep lingual groove was present in all mandibular premolars starting from mid-root till the apex providing a C-shape anatomy (Fig. 2). The axial cross-sections also revealed the presence of C-shaped root canal configuration in all mandibular premolars (Fig. 3). A C-shaped orifice in the coronal third divided into two or more discrete and separate canals in the mid-root to the apex. (Fig. 3). Such types of C-shaped canal configuration are classified as Category- III, Subdivision-II (1).

The morphology of the involved teeth obtained with spiral CT in transverse, axial, and sagittal sections along with three-dimensional reconstructed images helped us to diagnose the rare incidence of C-shaped root canal anatomy in mandibular premolars bilaterally.

## Discussion

Mandibular premolars are considered to be one of the most difficult teeth for endodontic treatment due to the presence of multiple root canals, apical deltas, and lateral canals. Apart from having multiple root canals, an important anatomic variation is the presence of C-shaped root canals in mandibular first premolars (4). The C-shaped canal variation of morphology is unusual and can lead to difficulties during treatment so the proper diagnosis of this situation is mandatory before treatment.

A preoperative radiograph and an additional radiograph from 15-20° mesial or distal projection can provide clues about the root canal morphology (7,8). The radiographic features of C-shaped roots are radicular fusion or proximity and a blurred image of a third canal in between (9). Conventional dental imaging techniques like periapical radiographs and panoramic imaging are easy, economical but they lack the perception in the third dimension. Short comings of conventional dental imaging are overcome by three dimensional imaging like CT and MRI. Recently Zheng *et al*. (3) used cone-beam computed tomography [CBCT] to evaluate the prevalence of C-shaped root canal systems in mandibular second molars. Cone-beam computed tomography [CBCT] is a noninvasive three-dimensional [3D] imaging technique, reported to be sufficiently precise for morphological analysis (10). Schwarz *et al*. (11) in 1987 introduced the technique of dental CT, also called Dentascan. CT data offer significant advances in the ability to reconstruct the tissues of the tooth before and after instrumentation and obturation which are fully retrievable for future evaluations. These imaging techniques have emerged as a powerful tool for evaluation of root canal morphology.

In the present case report, spiral CT was used as a diagnostic tool to identify the unusual and complex root canal configuration in mandibular first and second premolars bilaterally. In the past, spiral CT has been used successfully for the diagnosis and treatment of teeth with complex root canal system (12).

## Conclusions

Anatomical variations occur commonly in mandibular premolars. Thus a dentist should be aware of C-shaped canals while performing endodontic treatment in these teeth. One should always use the advanced diagnostic aids when there is any radiographic evidence of complex root canal configuration. Spiral CT plays an essential role to recognize unusual and complex root canal morphology and assist in formulating a better treatment plan.
